# Effect of palatal shape on dental arch relationships in unilateral cleft lip, alveolus, and palate

**DOI:** 10.1007/s00784-026-07063-8

**Published:** 2026-08-07

**Authors:** Marek Skotarski, Michał Urzędowski, Marcin Olek, Aleksandra Bogusz, Andrzej Brudnicki, Piotr S. Fudalej

**Affiliations:** 1 Department of Orthodontics, Jagiellonian University Medical College, Montelupich Str. 4, Kraków, 31-155 Poland; 2 Department of Maxillo-facial Surgery, Pediatric Surgery Clinic, Institute of Mother and Child, Kasprzaka Str. 17a, Warsaw, 01-211 Poland

**Keywords:** Cleft lip and palate, Dental arch relationship, Geometric morphometrics, GOSLON Yardstick, Palatal shape

## Abstract

**Introduction:**

Palatoplasty is a critical surgery for restoring structure and function in patients with cleft palate, but surgical scarring is considered a major factor in maxillary growth deficiency and abnormal dental arch relationships. Palatal scars can significantly alter palatal morphology. The aim of this study was to investigate the relationship between palatal shape and dental arch relationships in children with complete unilateral cleft lip, alveolus, and palate (CUCLAP).

**Methods:**

Palatal shape was analyzed using 239 landmarks on digital models of casts obtained from 50 children with CUCLAP (mean age 9.8 years, standard deviation [SD] 0.91 years; 34 boys) who underwent one-stage cleft repair at 8 months old (SD 2 months), and from 60 non-cleft controls (mean age 8.6 years, SD 1.2 years; 25 boys). Some CUCLAP patients had received simple orthodontic treatment prior to evaluation, and most had undergone alveolar bone grafting as part of their cleft management; the control group had no history of orthodontic treatment or bone grafting. Procrustes superimposition and principal component analysis were performed to characterize the sample; deviations in palatal shape were quantified as the Procrustes distance (ProcDist) from the average non-cleft palate. Dental arch relationships were scored twice with the GOSLON Yardstick by four assessors. A correlation analysis evaluated the relationship between the ProcDist and GOSLON score. Digitization error was assessed through repeat digitization of 25 UCLP cases. Kappa statistics were used to assess agreement among and within raters.

**Results:**

Inter-rater agreement for GOSLON score ranged from κ = 0.64 to 0.82 (lower limit 95% confidence interval = 0.61), and intra-rater agreement ranged from κ = 0.66 to 0.88. Digitization error accounted for 4.81% of total variability. The mean GOSLON score was 2.95 (SD 0.83). The ProcDist correlated with the GOSLON score (*R* = 0.28; *p* = 0.045).

**Conclusions:**

Palatal shape is associated with dental arch relationships in CUCLAP, and geometric morphometric assessment of palatal deviation may complement existing occlusal indices in the evaluation of treatment outcomes. When automated, such techniques may offer an objective and reproducible tool for quantifying the degree of palatal deviation from a normative shape.

**Supplementary Information:**

The online version contains supplementary material available at https://doi.org/10.1007/s00784-026-07063-8.

## Introduction

Management of cleft lip and palate (CLP) requires multidisciplinary care involving surgical, orthodontic, and speech interventions [1]. Despite continuous refinement of treatment protocols, maxillary growth disturbance remains a major clinical challenge, particularly in severe forms of CLP, such as unilateral CLP (UCLP) or bilateral complete CLP [2]. Growth deficiency is primarily attributed to scarring following palatoplasty [3]; the greater the degree of scar tissue formed after closure of the cleft, the higher the likelihood of maxillary hypoplasia. This deficiency may result in unfavorable dental arch relationships and compromised nasolabial esthetics.

In individuals with UCLP, dental arch relationships and palatal morphology provide complementary information on treatment outcome. Dental arch relationships reflect the sagittal and transverse coordination of the maxillary and mandibular arches - less favorable outcomes are typically characterized by maxillary arch constriction, anterior or posterior crossbite, reduced or reverse overjet, and a tendency toward skeletal Class III malocclusion. Palatal morphology, in turn, reflects the shape, width, symmetry, and vault configuration of the repaired maxillary arch, which in UCLP is often asymmetric and transversely constricted, particularly in the region of the cleft. To facilitate systematic patient categorization and support prediction of treatment complexity, several indices have been developed [4]. Among these, the GOSLON Yardstick [5] is likely the most widely used tool for evaluating occlusal outcomes in UCLP. GOSLON scores have been applied extensively in intercenter comparisons, such as Eurocleft [6], Americleft [7], and Scandcleft [8], demonstrating satisfactory reproducibility and acceptable, though not ideal, prognostic value for later orthodontic and surgical needs [9]. Although clinically useful, the GOSLON Yardstick and similar occlusal indices do not capture the underlying morphology of the palate, which can somewhat limit their prediction value. This weakness prompted the development of the Eurocran Index [10], which also includes evaluation of the palatal surface. However, visual assessment of palatal morphology has proven difficult to standardize: the palatal component of the EUROCRAN index - specifically developed to capture this dimension - demonstrated poor reliability and unproven validity when compared against established occlusal indices [11].

Evaluation of palatal morphology is important because the palate is the primary site of scar tissue formation following palatoplasty [12, 13]. A more precise method of evaluating palatal form, the anatomical region where maxillary growth restriction is initiated, could enhance growth prediction, improve treatment individualization, and guide refinement of surgical protocols. The latter is particularly compelling, as the earlier a surgical technique can be shown to induce more extensive scarring, the earlier modifications can be introduced. Existing indices do not provide sufficient precision for this level of assessment [14].

Evaluation of palatal shape using geometric morphometrics (GMs) [15] allows objective, detailed, three-dimensional (3D) assessment of palatal morphology and facilitates comparisons between groups. This approach has been successfully used to identify subtle variations in craniofacial skeletal morphology [16], facial form [17], and differences in palatal shape among patients with UCLP subjected to various treatment protocols [18]. However, the association between palatal morphology and occlusal outcomes in UCLP remains unclear.

The aim of the present study was to evaluate the relationship between palatal shape and dental arch relationships assessed using the GOSLON Yardstick. Specifically, we examined whether deviation of an individual’s palatal shape from the mean non-cleft palatal morphology correlates with their GOSLON scores.

## Materials and methods

This retrospective study was approved by the institutional Bioethics Committee (reference #21/2013).

### Sample

Palatal morphology was evaluated in children with complete unilateral cleft lip, alveolus, and palate (CUCLAP group), and in children without cleft deformity (control group). Dental arch relationships were assessed exclusively in the CUCLAP group.

#### CUCLAP group

All children in this group underwent one-stage closure of the complete cleft at the Institute of Mother and Child (IMC) in Warsaw, Poland, as described previously [19]. The inclusion criteria were non-syndromic status, receipt of all surgical treatments at the IMC, and availability of high-quality dental casts. The casts were taken at approximately 10 years of age. Patient eligibility was determined by the clinicians based on comprehensive diagnostic information obtained from medical records. None of the patients received presurgical (infant) orthopedic treatment.

One-stage primary cleft repair was performed in a single operation, typically between 6 and 9 months of age, and combined palatoplasty of both the hard and soft palate with cheiloplasty. The procedure began with repair of the soft palate. During this stage, all abnormal muscle insertions were dissected from the posterior edge of the hard palate to the hamuli, which were fractured. The palatal muscles were then reconstructed and sutured in the midline. This was followed by hard palate repair using an extended vomer flap, with tight closure of the anterior palate. The operation was completed with lip closure using a triangular flap. No infant orthopedics or primary nasal surgery was performed. Alveolar bone grafting (ABG) was performed in 41 subjects using a corticocancellous bone block harvested from the anterior iliac crest, inserted firmly between the bony edges of the alveolar cleft with the cortical lamina facing the labial side; oronasal fistulas, when present, were closed at the same time. Thirty-three subjects underwent early ABG at 2–4 years of age and eight underwent ABG at 7–9 years. All cleft surgeries were performed at IMC by a team of five surgeons, with the three most experienced performing all one-stage UCLP repairs using consistent surgical techniques regardless of patient age or operator. In subjects who had undergone ABG, dental casts were obtained at least 1.5 years after the procedure. For further details on ABG see Supplementary material.

Orthodontic treatment comprised maxillary expansion to correct posterior cross-bites. Removable appliances, such as Schwarz’s plates, were used in the majority of patients. Maxillary expansion was typically initiated before 6 years of age. The removable plates were retained after active expansion to maintain the stability of the maxilla until alveolar bone grafting could be performed. Quad-helix expanders were occasionally used. Fixed appliances were used only in cases in which central incisor malalignment needed to be corrected during this phase. Facial masks or skeletal anchorage were not incorporated into the treatment.

#### Control group

The control group comprised children seeking orthodontic consultation based on the following inclusion criteria: good health, Class I malocclusion, and no history of prior orthodontic treatment. Children with cross-bite, signs of multiple and/or advanced caries, tooth agenesis, or observable supernumerary teeth were excluded. The control group also did not include children with cleft lip and/or palate or other congenital syndromes within craniofacial structures.

### Evaluation of palatal shape

**Fig. 1:**
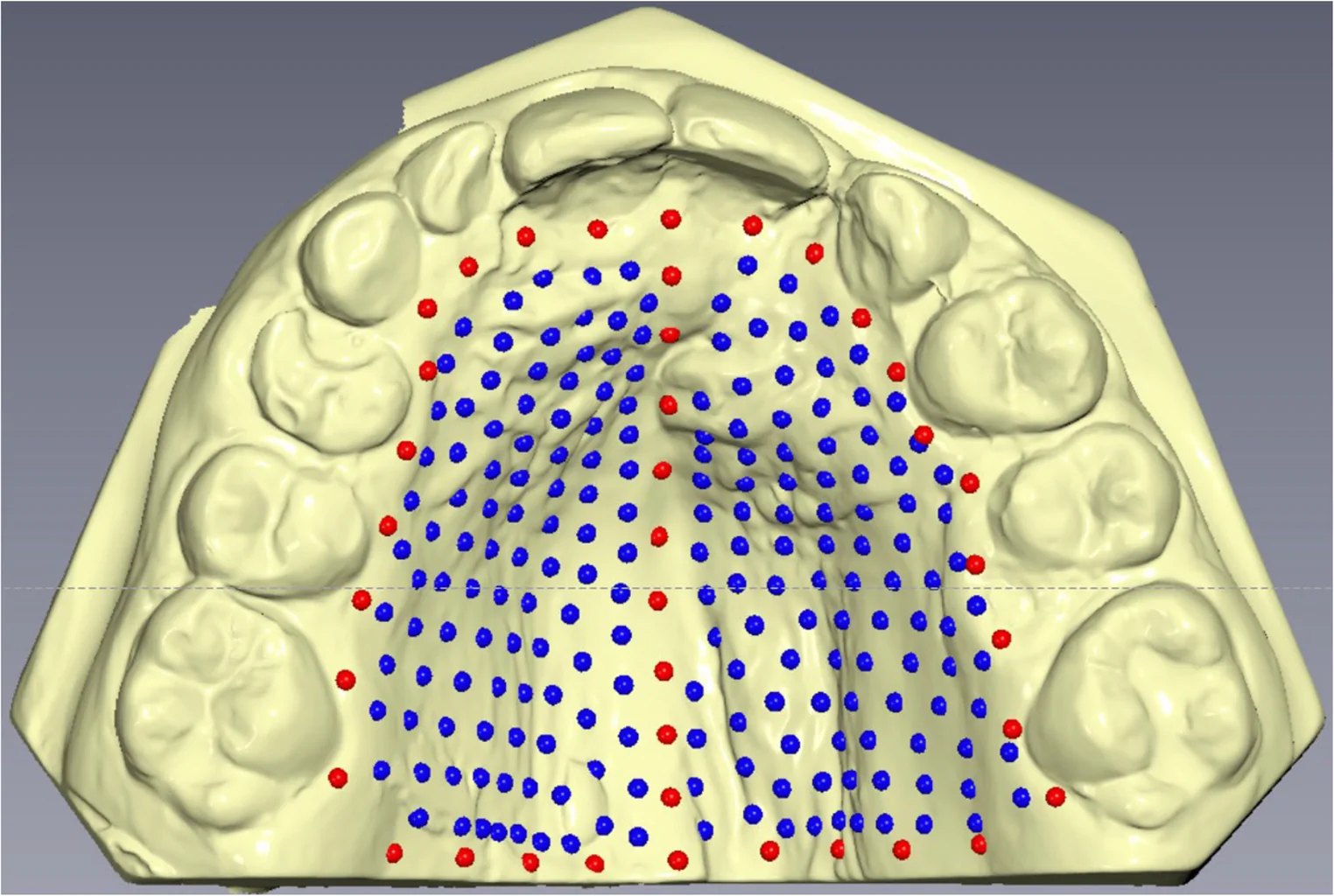
Graphical representation of the landmarks placed on the palatal surfaces of digital casts. Fixed landmarks are in red and semi-landmarks in blue. (Reproduced from Petrova et al. (Journal of Clinical Medicine, 2023, doi: https://doi.org/10.3390/jcm12185985) under the Creative Commons Attribution 4.0 International License (CC BY 4.0))

The Trios 3 intraoral scanner (3Shape A/S, Copenhagen, Denmark) was used to digitize plaster casts of the maxilla as STL files. When the cleft was situated on the right side, the scan was horizontally mirrored to consistently portray the cleft on the left side. The Viewbox 4 program (dHAL software, Kifissia, Greece) was used to identify 239 landmarks on each digital model (Fig. 1). Initially, 39 „fixed“ landmarks were manually placed on the palatal surface: 9 points along the midsagittal line (from the incisive papilla to its intersection with the posterior curve), 9 points along a posterior curve perpendicular to the midsagittal line (from the distal aspect of the upper left to the upper right first permanent molar), and 21 points outlining the dental arch perimeter apical to the gingival sulci (from the palatal groove of the upper left first permanent molar, through the midpoint between the upper central incisors, to the palatal groove of the upper right first permanent molar). In each series, intermediate landmarks were distributed at equal intervals between the anchor points.

The remaining landmarks (“semi-landmarks”) were automatically positioned uniformly on the palatal surface within the boundaries defined by the fixed landmarks. To ensure homologous positioning of all landmarks across subjects, the semi-landmarks were subjected to a sliding-projecting process three times. This process involved adjusting the landmarks to minimize bending energy, projecting them back onto the palatal surface, and then further adjusting them through sliding.

### Assessment of dental arch relationships

Dental arch relationships were evaluated on digital models by four calibrated raters (PF, MO, MS, MU) using the GOSLON Yardstick. Each rater independently scored all models in two separate sessions, with case order randomized between sessions. Raters were blinded to participant age, sex, and morphometric outcomes. Reference GOSLON models representing all five categories were available throughout the scoring process, and no time limits were imposed.

### Reliability

The reliability of the digitization was assessed by repeating landmark placement on a randomly selected subset of 25 models after an interval of at least 1 month. Discrepancies between the repeated digitizations were quantified by calculating Procrustes distances (ProcDist) between digitizations. The ProcDists were compared with the overall shape variance in the sample to estimate method error.

The reliability of GOSLON scoring was evaluated by calculating inter-rater and intra-rater agreement. Linear weighted Cohen’s kappa coefficients with 95% confidence intervals were computed and interpreted according to the guidelines proposed by Landis and Koch [20].

### Statistical analysis

Palatal shape was analyzed by GM techniques. We performed generalized Procrustes superimposition of homologous landmark configurations, followed by principal component analysis (PCA) to reduce data dimensionality while retaining critical features. The average palatal shape was determined in the control group by calculating the mean Procrustes coordinates for all landmarks. ProcDists between the average non-cleft shape and the palatal shape for each patient in the CUCLAP group were computed to measure deviation. A greater distance indicated more deviation.

The average GOSLON scores were calculated across the four raters. Pearson’s correlation coefficient was used to examine the association between the ProcDist for each patient in the CUCLAP group (i.e. the deviation from the average non-cleft shape) and their corresponding mean GOSLON rating. Spearman’s correlation was additionally computed as a sensitivity analysis. All correlation analyses were carried out using PAST software v. 4.17 [21]. Significance was defined as *p* < 0.05.

## Results

### Demographic characteristics

The CUCLAP group comprised 50 children, including 34 boys (68%) and 16 girls (32%). The mean age at the time the diagnostic models were made was 9.8 years (standard deviation [SD] 0.91 years; range 8.0–13.6 years). The control group comprised 60 children (25 boys and 35 girls) with a mean age of 8.6 years (SD 1.2 years). As palatal shape was comparable between girls and boys [18], regardless of the presence of a cleft, sexes were combined in the analysis.

Of the 50 patients, 48 (96%) were in the mixed dentition and 2 (4%) in the permanent dentition at the time of model acquisition. A missing lateral incisor was recorded in 34 patients (68%) and a missing canine in 19 patients (38%); only 6 patients (12%) had no missing teeth recorded on the models. For details see Supplementary material.

### Method error

The mean error of the method accounted for 4.8% of the total variance in shape. Investigation of the intra-rater and inter-rater agreement (Table 1) indicated substantial to almost perfect agreement.

**Table 1:** Assessment of rater agreement in scoring dental arch relationships with the GOSLON Yardstick in the CUCLAP group

Rater(s)	Agreement	Expected agreement	Kappa (LL 95% CI)
PF vs. MO	91.98%	75.77%	0.67 (0.64)
PF vs. MS	92.92%	76.03%	0.70 (0.67)
PF vs. MU	93.40%	73.00%	0.76 (0.73)
MO vs. MS	94.97%	71.52%	0.82 (0.79)
MO vs. MU	91.04%	75.16%	0.64 (0.61)
MS vs. MU	92.92%	75.68%	0.71 (0.68)
MS (intra-rater)	92.00%	31.20%	0.88 (0.85)
PF (intra-rater)	76.00%	30.4%	0.66 (0.64)
MU (intra-rater)	80.00%	26.2%	0.73 (0.72)
MO (intra-rater)	80.00%	33.8%	0.82 (0.77)

*LL* lower limit, *CI* confidence interval

### Procrustes superimposition and PCA

**Fig. 2:**
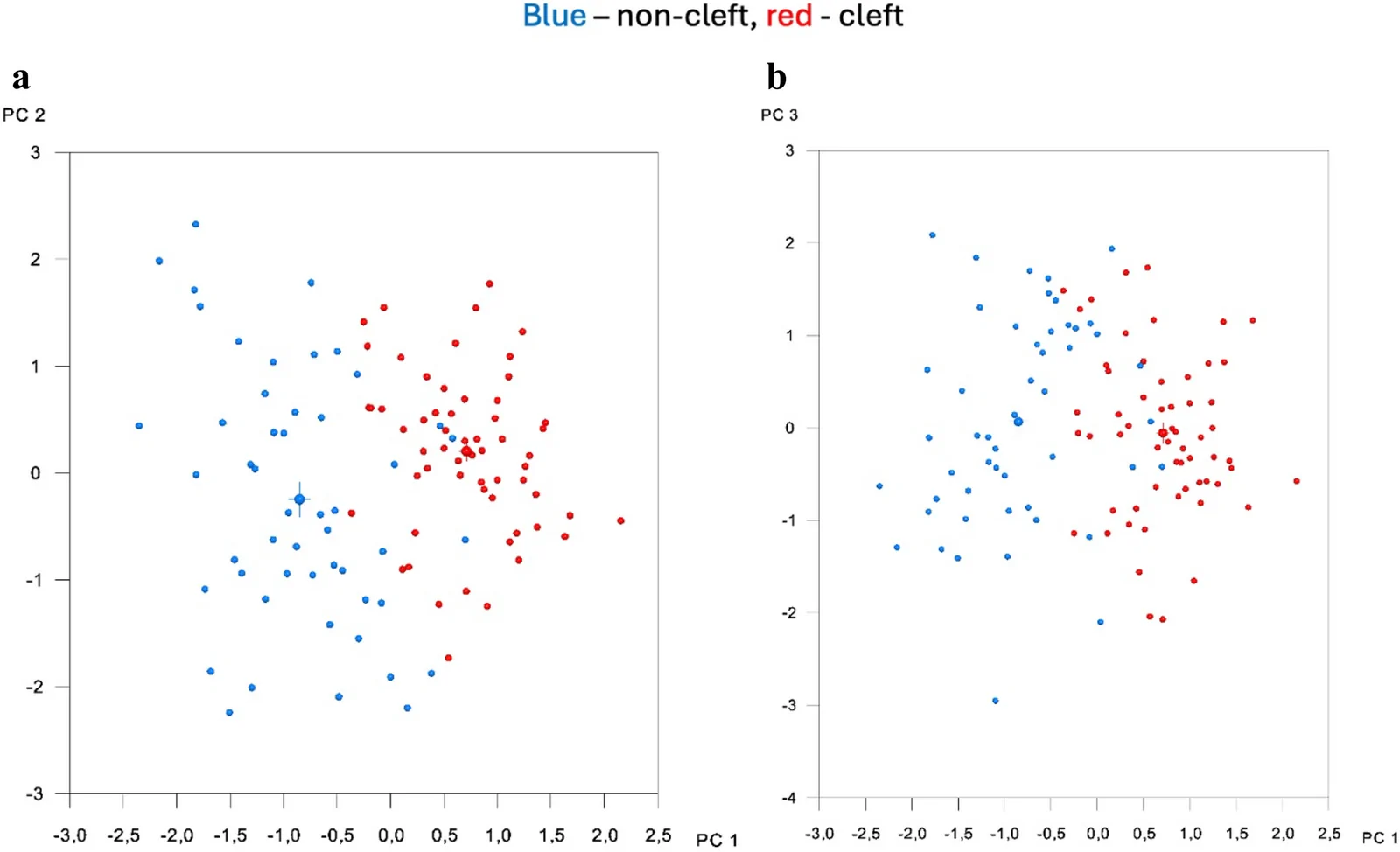
Distribution of individual participants (smaller circles) and group averages (larger circles) in the shape space described by (**a**) principal components (PCs) 1 and 2 and (**b**) PCs 1 and 3. Blue indicates the control group and red the CUCLAP group

The initial 22 principal components (PCs) captured non-trivial variation in shape, collectively accounting for 92.5% of the overall shape variability (Supplementary material available online). The first six PCs contributed to at least 5% of the total shape variability: PC1 = 30%, PC2 = 10.9%, PC3 = 9%, PC4 = 7.9%, PC5 = 7.2%, and PC6 = 5% (69.9% in total). The distribution of individual participants is visually depicted in Fig. 2.

The Supplementary material also illustrates that PC1 primarily characterized morphological variations in palate width and length, displaying noticeable patterns of wide and short or narrow and long. Furthermore, PC1 revealed variations related to the side of the cleft. PC2 predominantly reflected variations in palatal height, whereas PC3 indicated cleft side-related variations across all three dimensions.

### Palatal shape and GOSLON scores

Table 2 summarizes the mean ProcDist (range: 0.107–0.246) and GOSLON scores across raters. The overall mean GOSLON score of 2.95 (SD 0.83) indicates intermediate occlusal outcomes. Neither sex nor age showed an association with GOSLON scores (*p* = 0.82 and *p* = 0.92, respectively). The distribution of GOSLON grades is provided in Supplementary material.

**Table 2:** Mean GOSLON Yardstick scores for individual raters and mean Procrustes distance (ProcDist) between the average non-cleft shape and each patient‘s palatal shape

	Mean	SD	P25	P75
PF	2.91	0.99	2	3
MO	2.85	0.82	2	3
MS	2.89	0.80	2	3
MU	3.02	1.01	2	4
ProcDist	0.173	0.036	0.148	0.198

*SD* standard deviation, *P25* 25th percentile, *P75* 75th percentile

**Fig. 3:**
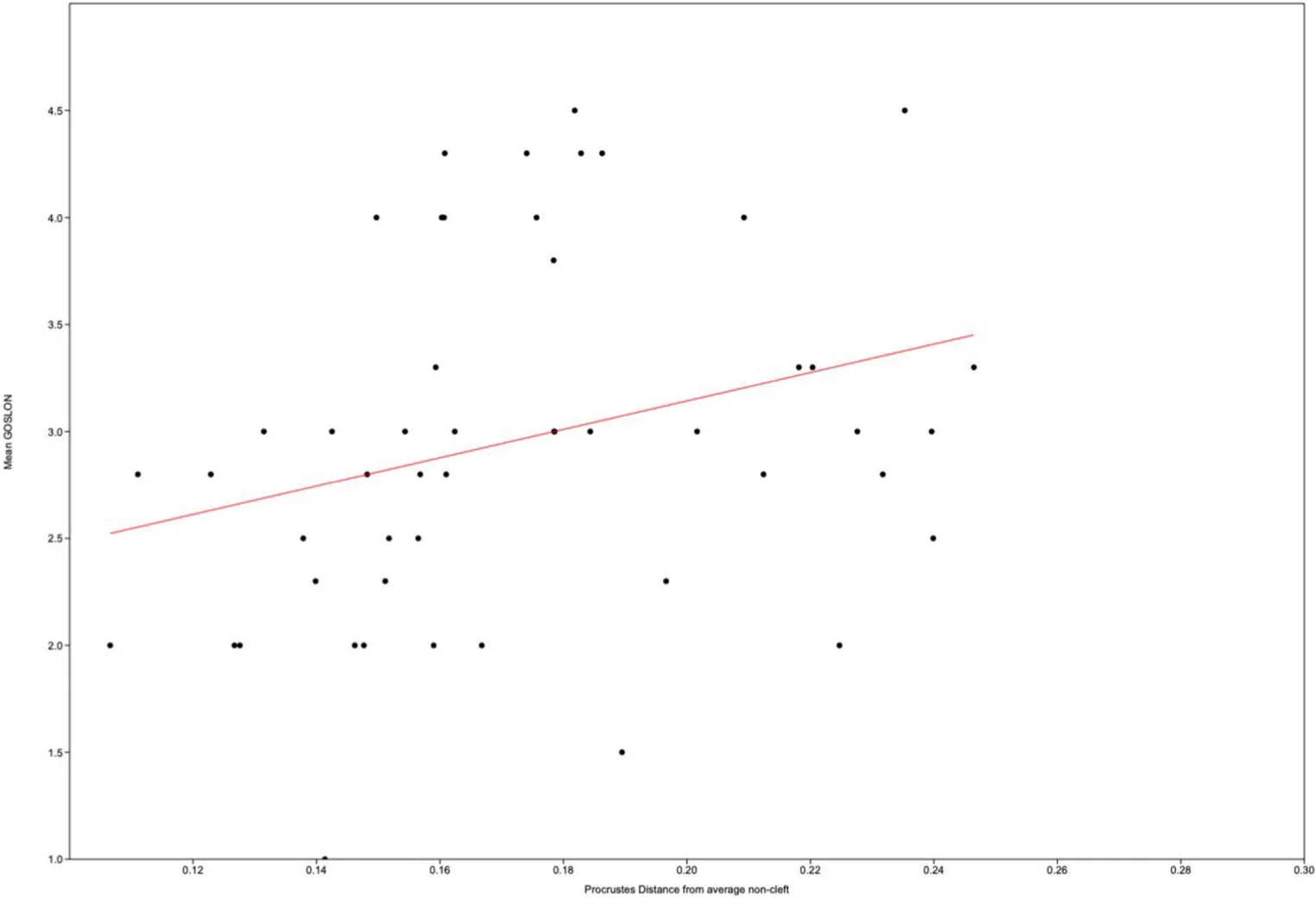
GOSLON Yardstick scores vs. distance from the average non-cleft shape. A regression line was fitted (in red)

A significant correlation between the ProcDist and the mean GOSLON score (Pearson’s *r* = 0.284, 95% CI [0.007, 0.521], *p* = 0.045; Spearman’s *r* = 0.391, 95% CI [0.126, 0.603], *p* = 0.005) suggests that greater morphological deviation is associated with poorer dental arch relationships (Fig. 3).

## Discussion

Predicting growth outcomes in CLP, such as maxillary deficiency or future dental arch relationships, is difficult because craniofacial growth is highly variable and influenced by many interacting factors, including inherent growth impairment and the unpredictable effects of surgical scarring. Variations in cleft severity, anatomy, surgical techniques, and treatment protocols add further complexity. Moreover, maxillary growth is nonlinear and patient-specific, with treatment effects interacting with natural development. Nevertheless, a reliable predictive method would greatly facilitate optimization of CLP management.

We found an association between the degree of deviation in palatal morphology, measured by the ProcDist, and dental arch relationships assessed with the GOSLON Yardstick - the more deviated the palatal shape, the poorer the dental arch relationship. The modest strength of this correlation can be interpreted from two complementary perspectives. On one hand, a relatively low correlation suggests a limited predictive value of palatal morphology alone, as < 10% of the variance in GOSLON scores could be explained by variation in the deviation in palatal shape. Following this reasoning, one might assume that a correlation coefficient approaching 1 would be ideal. However, such a scenario would imply that the two variables are nearly redundant, offering little additional information and making one of them potentially unnecessary. Therefore, a perfect correlation is neither realistic nor desirable. On the other hand, the absence of any correlation would indicate that a deviation in palatal shape is unrelated to maxillary growth deficiency, which is indirectly reflected by the GOSLON score. From this perspective, the observed moderate correlation can be viewed positively as indicating that palatal morphology is meaningfully associated with dental arch relationships and, thus, with maxillary growth. When combined with the GOSLON Yardstick or other indices, assessment of palatal shape may provide additional information on dental arch relationships in CLP, although longitudinal studies are needed to determine whether early palatal shape deviation has predictive value for later growth or treatment outcomes.

Quantifying the deviation of an individual cleft palate shape from an average non-cleft shape using ProcDist is a relatively new approach but has shown considerable promise in the precise assessment of morphological differences. Direct measurements were the first techniques applied to evaluate 3D palatal morphology in CLP [22–24]. Although these measurements are simple to perform, they cannot capture the full complexity of the palatal form, particularly in the presence of palatal asymmetry as in CLP. Advances in plaster cast digitization and the introduction of direct intraoral scanning have enabled high-resolution 3D reconstruction of the palate and more sophisticated morphometric analyses, including the quantification of palatal surface area and volume [25–27]. Color-coded distance maps, which have strong visual appeal, are frequently used to illustrate surface differences [28, 29] but reflect distances between the nearest points on two surfaces, which may not be anatomically homologous. Thus, such maps may misrepresent the true morphological variations or changes. In contrast, GM techniques preserve anatomical correspondence among landmarks and surface points, enabling more accurate comparisons [30]. Efforts have also been made to automate the analysis of 3D scans [31, 32]. Though our GM-based approach is not yet fully automated, it comprehensively evaluates palatal morphology by calculating the distance between a reference (i.e., the shape of an average non-cleft palate) and individual cleft shapes, providing a precise measure of both the overall deviation in shape and the asymmetry in cleft-affected regions. However, the use of a new method limits comparisons with the results of other studies, as our assessment of palatal shape cannot be directly compared with linear measurements or with evaluations of surface area or volume.

PCA showed that the CUCLAP and control groups differed most markedly in terms of palatal width and length. Patients in the CUCLAP group had narrower and shorter palates than controls. This is illustrated by the visualization of variability for PC1 and is consistent with previous reports. Using comparable methods, Rusková et al. [33] showed that PC1 accounted for 31% of the total variance in palatal shape among cleft and non-cleft subjects, with the primary differences being related to transverse width and palatal asymmetry, closely matching both the magnitude and general direction of our PC1 (~ 30%). A study in mixed dentition [25] reported reduced palatal surface area and volume in patients with cleft compared with non-cleft controls. Similar conclusions have been drawn from conventional cast measurements, which consistently demonstrate smaller maxillary arch widths and lengths in individuals with UCLP compared with controls [34]. In contrast, a study from Spain [35] did not identify significant differences in intercanine width or arch depth between cleft and non-cleft groups, though palatal volume was reduced in the cleft cohort. These discrepancies may reflect differences in sample characteristics, including assessments in young adults and the potential influence of prior orthodontic expansion.

Some study participants had undergone simple orthodontic treatment, mainly with Schwartz plates, prior to evaluation. Additionally, not all permanent teeth had erupted at the time of assessment - either due to congenital agenesis or because evaluation occurred after exfoliation of a primary tooth but before eruption of its permanent successor; the nature of the missing tooth could be determined from the available records. Orthodontic treatment can temporarily shift dental arch relationships toward more favourable GOSLON categories without reflecting the true surgical outcome [36], an effect that is amplified in patients with poorer arch relationships whose scores are inherently less stable over time [37]. At the same time, orthodontic treatment cannot compensate for adverse surgical outcomes — GOSLON rankings in the two poorest-performing Eurocleft centers showed no improvement despite prolonged treatment [6]. Dental agenesis, in turn, should be regarded not as a confounding variable but as a marker of underlying severity, with missing lateral incisors found significantly more often in GOSLON categories 4–5 than in categories 1–2 [38]. Importantly, lateral incisor agenesis alone was not associated with significantly worse scores - it was only the presence of two or more missing maxillary teeth that was associated with a substantially worse GOSLON distribution and greater skeletal Class III tendency [39].

The cleft deformity in the CUCLAP group was repaired using a one-stage protocol, meaning that all cleft structures were closed during a single operation, which was performed between 6 and 9 months of age. This procedure, carried out by highly experienced surgeons, began with soft palate repair and concluded with lip repair. Globally, such an approach is rarely used, as most surgical protocols involve two or three separate procedures. One might argue that using a sample treated with a less common protocol limits the generalizability of our findings. However, The Scandcleft trials [40] did not demonstrate a clear superiority of any single primary surgical protocol in terms of favorable or unfavorable outcomes. Rather, the results should be interpreted in light of surgeon-related factors, including annual case volume, technical experience, and familiarity with the assigned protocol, since surgery may be more challenging when surgeons are required to use a technique with which they have limited prior experience.

Combined with the relatively good occlusal outcomes observed in our sample compared with other cleft centers, these observations suggest that the one-stage repair method used in our cohort allows for reasonable generalizability, albeit primarily to high-volume settings with experienced surgeons. Importantly, the patients in our sample included the full range of GOSLON scores, representing both favorable and unfavorable dental arch relationships. This broad distribution of outcomes is essential for examining the relationship between palatal shape and dental arch morphology, as it ensures that the entire spectrum of occlusal results is captured.

### Limitations

The findings of this study should be interpreted with caution in view of several important limitations. First, the cross-sectional design captures palatal morphology and occlusal relationships at a single age and, therefore, cannot account for growth-related changes or long-term treatment outcomes. Second, although ProcDist was associated with the GOSLON score, the strength of association was relatively weak, underscoring that palatal shape is just one of many factors influencing occlusal development. In addition, although the GOSLON Yardstick demonstrated good reliability in this study, it remains an ordinal and partially subjective measure, and its inherent limitations should be kept in mind. Third, the control group was approximately 1.2 years younger on average than the CUCLAP group (8.6 vs. 9.8 years). Since the average non-cleft palatal shape - used as the reference for all ProcDist calculations - was derived from the control group, any age-related differences could systematically affect the ProcDist values computed for CUCLAP patients. However, available evidence suggests that dimensional changes (*shape* alterations have not been assessed) during this interval are relatively limited [41, 42] suggesting that the practical impact of this discrepancy on our findings is likely small. Additionally, the exclusion criteria for the control group did not explicitly address mouth breathing status, as this information was not systematically recorded. Since mouth breathing may alter palatal morphology, its potential presence in the control group could have influenced the reference shape used for ProcDist calculation. Finally, both the CUCLAP and control cohorts were drawn from a single Central European population, which may restrict the generalizability of the findings in populations of different ethnic or geographic backgrounds.

## Conclusions

Palatal shape was associated with dental arch relationships in children with CUCLAP. Geometric morphometric techniques, particularly when automated, may provide effective tools for quantifying the degree of palatal shape deviation. Further research is warranted to examine the effects of different surgical protocols, ideally in larger, multicenter, longitudinal cohorts.

### Clinical relevance

The present method offers a potential clinical application as an objective, continuous descriptor of palatal morphological deviation, complementing established occlusal indices such as the GOSLON Yardstick. Integration with automated scoring pipelines - including automated GOSLON assessment - could in the future facilitate longitudinal monitoring and inter-center comparisons. 

## Supplementary Information

Below is the link to the electronic supplementary material.

Supplementary Material 1 (DOCX 676 KB)

## Data Availability

Data files can be obtained from the corresponding author upon reasonable request.
