# Supplementary material for: Effect of palatal shape on dental arch relationships in unilateral cleft lip, alveolus, and palate
Source: Clin Oral Investig. 2026 Aug 7;30(9):372. doi: 10.1007/s00784-026-07063-8 (PMC13447454; doi:10.1007/s00784-026-07063-8)
Supplement: Supplementary file 1 [file 784_2026_7063_MOESM1_ESM.docx]

**Supplementary material**

1. **Alveolar bone grafting (ABG) in the sample**

|  |  | Age in years at ABG | Age in years when models were made | | Temporal distance between ABG and models |
| --- | --- | --- | --- | --- | --- |
| All who had ABG (N = 41) | Min | 1.4 | 8.0 | | 1.5 |
|  | Max | 8.7 | 13.6 | | 9.2 |
|  | Mean | 3.3 | 9.8 | | 6.4 |
|  | Stand. dev | 2.4 | 1.0 | | 2.1 |
|  | Median | 2.2 | 9.9 | | 7.0 |
|  | 25 prcntil | 2.0 | 9.2 | | 5.8 |
|  | 75 prcntil | 3.6 | 10.1 | | 7.9 |
| Those who had early ABG (N = 33) | Min | 1.4 | 8.0 | | 4.8 |
|  | Max | 4.1 | 11.3 | | 9.2 |
|  | Mean | 2.2 | 9.6 | | 7.3 |
|  | Stand. dev | 0.6 | 0.8 | | 1.0 |
|  | Median | 2.1 | 9.8 | | 7.3 |
|  | 25 prcntil | 1.9 | 8.9 | | 6.8 |
|  | 75 prcntil | 2.3 | 10.1 | | 8.1 |
| Those who had late ABG (N = 8) | Min | 7.2 | 9.7 | | 1.5 |
|  | Max | 8.7 | 13.6 | | 5.4 |
|  | Mean | 8.0 | 10.6 | | 2.7 |
|  | Stand. dev | 0.6 | 1.3 | | 1.2 |
|  | Median | 8.0 | 10.2 | | 2.4 |
|  | 25 prcntil | 7.4 | 9.9 | | 1.8 |
|  | 75 prcntil | 8.5 | 10.8 | | 3.0 |
|  |  |  |  |  | |
|  |  | early ABG < 5 years |  |  | |
|  |  | late ABG > 7 years |  |  | |

2. **Dentition stage of study participants at the time of cast acquisition.**

| **Tooth type** | **Patients with any missing (n)** | **Proportion of the sample (%)** |
| --- | --- | --- |
| Central incisor | 0 | 0% |
| Lateral incisor | 34 | 68% |
| Canine | 19 | 38% |
| 1st premolar OR 1st deciduous molar | 20 | 40% |
| 2nd premolar OR 2nd deciduous molar | 19 | 38% |
| 1st molar | 0 | 0% |

3. **Distribution of GOSLON grades in the sample averaged for four assessors**

| **Range of averaged scores** | | **Number of subjects within the range of GOSLON score** |
| --- | --- | --- |
| 1 | 1.25 | 1 |
| 1.26 | 1.5 | 0 |
| 1.51 | 1.75 | 1 |
| 1.76 | 2 | 0 |
| 2.01 | 2.25 | 8 |
| 2.26 | 2.5 | 3 |
| 2.51 | 2.75 | 4 |
| 2.76 | 3 | 7 |
| 3.01 | 3.25 | 10 |
| 3.26 | 3.5 | 4 |
| 3.51 | 3.75 | 0 |
| 3.76 | 4 | 1 |
| 4.01 | 4.25 | 5 |
| 4.26 | 4.5 | 6 |
| 4.61 | 5 | 0 |

4. ***Variation in the sample explained by non-trivial principal components (PCs)***

|  | **Variance** | **Cumulative variance** | **Broken-stick criterion** |
| --- | --- | --- | --- |
| PC 1 | 30.0% | 30.0% | 1.0% |
| PC 2 | 10.9% | 40.8% | 0.9% |
| PC 3 | 9.0% | 49.8% | 0.8% |
| PC 4 | 7.9% | 57.7% | 0.7% |
| PC 5 | 7.2% | 64.9% | 0.7% |
| PC 6 | 5.0% | 69.9% | 0.7% |
| PC 7 | 3.9% | 73.8% | 0.7% |
| PC 8 | 2.7% | 76.5% | 0.6% |
| PC 9 | 2.3% | 78.9% | 0.6% |
| PC 10 | 2.0% | 80.8% | 0.6% |
| PC 11 | 1.8% | 82.6% | 0.6% |
| PC 12 | 1.4% | 84.0% | 0.6% |
| PC 13 | 1.3% | 85.3% | 0.6% |
| PC 14 | 1.2% | 86.5% | 0.6% |
| PC 15 | 1.1% | 87.6% | 0.5% |
| PC 16 | 1.0% | 88.6% | 0.5% |
| PC 17 | 0.8% | 89.4% | 0.5% |
| PC 18 | 0.8% | 90.2% | 0.5% |
| PC 19 | 0.7% | 90.9% | 0.5% |
| PC 20 | 0.6% | 91.4% | 0.5% |
| PC 21 | 0.6% | 92.0% | 0.5% |
| PC 22 | 0.5% | 92.5% | 0.5% |

5. ***Graphic depiction of the first three principal components (PCs) of the palate from the three views. Red lines indicate −3 standard deviation and blue lines +3 standard deviation.***
